# Targeting FOXO3 for the Management of Heart Failure: Current Evidence and Future Directions

**DOI:** 10.3390/ph19091460

**Published:** 2026-09-15

**Authors:** See-Hyoung Park

**Affiliations:** Department of Biological and Chemical Engineering, Hongik University, Sejong 30016, Republic of Korea; shpark74@hongik.ac.kr; Tel.: +82-44-860-2126

**Keywords:** heart failure, cardiomyocytes, FOXO3, phytochemicals, pharmaceuticals

## Abstract

Heart failure (HF) is a multifaceted clinical syndrome marked by the heart’s inability to pump sufficient blood, resulting in symptoms such as dyspnea, fatigue, and fluid retention. Among the emerging therapeutic strategies for managing HF, targeting the transcription factor forkhead box O3 (FOXO3) has garnered attention due to its regulatory role in cellular processes related to oxidative stress, apoptosis, autophagy, and inflammation. Dysregulation of FOXO3 has been implicated in the progression of HF, making it a notable focus for potential therapeutic interventions aimed at improving cardiac function and patient outcomes. Research into FOXO3-targeting compounds has identified various classes of agents, including phytochemicals and pharmaceuticals. These compounds work through mechanisms such as interaction with upstream signaling pathways and downstream target genes of FOXO3. While preclinical studies show promise for FOXO3 targeting compounds in promoting cardiac health and function, challenges remain in translating these findings into successful clinical applications. In this review article, we focus on the FOXO3 modulators investigated between 2006 and 2026 that have shown beneficial effects on heart function in diverse experimental models. Furthermore, we discuss current methodological limitations in this research field and propose potential directions for future studies on heart health management.

## 1. Introduction

### 1.1. Recent Trends of Heart Failure

The definition and classification of heart failure (HF) follows Second Universal Definition of Heart Failure (2026), jointly issued by the AHA, ACC, ESC, and WHF [1]. HF is a clinical syndrome characterized by current or prior symptoms and/or signs caused by a structural and/or functional cardiac abnormality, corroborated by elevated natriuretic peptide levels and/or objective evidence of pulmonary or systemic congestion. HF progresses through a four-stage continuum (Stages A–D). Stage A includes individuals at risk for HF without symptoms, structural cardiac changes, or elevated biomarkers. Stage B (pre-HF) comprises asymptomatic individuals with structural or functional cardiac abnormalities or elevated natriuretic peptides or troponins. Stage C denotes symptomatic HF and Stage D represents advanced HF with severe symptoms despite guideline-directed therapy and consideration of advanced interventions. In addition to staging, the 2026 ESC Guidelines for the management of HF eliminated the intermediate HF with the mildly reduced ejection fraction (HFmrEF) category and now define only two phenotypes based on a single left ventricular ejection fraction (LVEF) cut-off of 50%: HF with reduced ejection fraction (HFrEF) with LVEF < 50% and HF with preserved ejection fraction (HFpEF) with LVEF ≥ 50% [2,3]. Each phenotype exhibits distinct primary pathophysiological drivers and underlying structural mechanics. HF has become a major global public health problem, with rising prevalence and persistent morbidity and mortality despite therapeutic advances. In 2021, an estimated 56.5 million people worldwide were living with HF, reflecting rapid growth in case numbers in parallel with population aging [4,5]. From a global perspective, HF contributes substantially to cardiovascular disease (CVD)-related hospitalizations, health-care costs, and premature death, even though it represents only one component of the broader CVD spectrum [6]. CVDs collectively account for roughly one-third of all deaths worldwide, and HF frequently represents the final common pathway of many upstream conditions, including ischemic heart disease, hypertension, and valvular heart disease [7]. Despite improvements in pharmacologic and device therapies, the long-term prognosis of HF remains poor, with mortality declining less steeply than for other major CVDs in recent decades. Consequently, HF imposes a disproportionate economic burden on health-care systems, with inpatient care for HF and related cardiorenal complications representing one of the highest cost categories within CVD. Thus, HF represents an evolving disease characterized by rising prevalence and sustained clinical and economic burden at both global levels [8,9]. Aging populations, increasing cardiometabolic risk, and the growing contribution of HF with preserved ejection fraction suggest that the HF burden will continue to expand in coming decades unless effective prevention and population-level risk-factor control strategies are widely adopted. Thus, there is still a need for comprehensive and multidisciplinary HF programs that integrate early detection, optimal medical therapy, device-based interventions, and community-level prevention to mitigate the rising societal impact of HF.

### 1.2. Mechanism of HF

HF represents a multifaceted clinical syndrome where the heart fails to pump adequate blood to satisfy the body’s metabolic demands, resulting in symptoms including shortness of breath, exhaustion, and edema [10]. The pathophysiology of HF involves several interrelated mechanisms, primarily affecting myocardial function and systemic circulation. Decreased cardiac output triggers persistent sympathetic nervous system activation [11]. Excessive catecholamine release increases heart rate and myocardial contractility, initially maintaining perfusion. However, chronic stimulation accelerates β-adrenergic receptor desensitization, promotes cardiomyocyte apoptosis, and drives cardiotoxicity and lethal arrhythmias. An imbalance between matrix metalloproteinases and tissue inhibitors of metalloproteinases disrupts extracellular matrix homeostasis [12]. Enhanced collagen deposition and cardiac fibroblast activation cause interstitial fibrosis, reducing ventricular compliance (diastolic dysfunction) and predisposing the myocardium to progressive dilatation (systolic failure). The failing heart undergoes a metabolic shift from fatty acid oxidation to glucose utilization, but mitochondrial dysfunction and impaired substrate oxidation ultimately lead to severe ATP deficiency [13]. Mitochondrial dysfunction and excessive generation of reactive oxygen species (ROS) further compromise energy production, driving structural decay and functional exhaustion of cardiomyocytes. In failing heart, endothelial dysfunction is characterized by reduced nitric oxide bioavailability, increased oxidative stress, and inflammation, which impair vascular regulatory capacity and contribute to disease progression and worse prognosis [14]. The activation of neurohormonal systems, particularly the renin–angiotensin–aldosterone system (RAAS), plays a pivotal role in the development and progression of HF. When activated, the RAAS leads to sodium and water retention, vasoconstriction, and myocardial remodeling, exacerbating the heart’s workload and contributing to deterioration in function [10,15]. Ischemia and reperfusion injury significantly impact myocardial health. This type of injury causes cardiomyocyte death and activates inflammatory pathways, leading to an increase in inflammatory cytokines. This inflammatory response can perpetuate tissue damage, resulting in a cycle of further injury and HF progression. Chronic inflammation also contributes to ventricular remodeling and deterioration of cardiac function [16]. Another critical aspect of HF is the impairment of angiogenesis, which affects the heart’s ability to form new blood vessels in response to injury. Insufficient angiogenic signaling, due to reduced levels of growth factors such as vascular endothelial growth factor (VEGF), leads to inadequate blood supply to the myocardium, worsening myocardial hypoxia and promoting the transition from hypertrophy to decompensation [17]. Mitochondrial dysfunction and oxidative stress are recognized as key contributors to the progression of HF. Abnormalities in mitochondrial respiration can lead to increased ROS production, which damages cardiomyocytes and exacerbates inflammation. These processes can influence cellular metabolism and energy production, ultimately impairing cardiac function [18]. These interconnected pathological mechanisms underscore the urgent need for novel therapeutic strategies that target specific pathways in HF treatment, promising to break the vicious cycle of progressive cardiac decompensation and improve patient outcomes beyond current standard-of-care limitations.

### 1.3. Pharmacological Therapy in HF

HF is a life-threatening condition in which rapid intervention is essential, and pharmacological therapy plays a central role in both acute stabilization and long-term management. Current standard treatment relies on a combination of chemical drugs, including renin angiotensin system blockers (ACEI/ARB), angiotensin receptor neprilysin inhibitors (ARNI), beta-blockers, mineralocorticoid receptor antagonists (MRA), and more recently sodium glucose cotransporter-2 inhibitors (SGLT2I), which reduce ventricular afterload and preload, modulate neurohormonal activation, and attenuate myocardial remodeling, thereby improving symptoms, lowering hospitalization rates, and extending survival [19]. Pharmacological strategies for HF vary according to LVEF. Across phenotypes, SGLT2I (dapagliflozin or empagliflozin) serve as the foundational, cross-phenotype therapy to reduce cardiovascular death and HF hospitalizations. Diuretics can be used in patients with HFpEF and HFrEF who have volume overload. For HFrEF, treatment centers on foundational quadruple therapy consisting of ARNI, ß-blocker (nebivolol, carvedilol, metoprolol succinate, or bisoprolol), MRA (spironolactone or eplerenone), and an SGLT2I. ACEI/ARB can be used when ARNI is not appropriate or tolerated. Digoxin as cardiac glycosides that inhibit Na^+^/K^+^-ATPase can be considered in patients with HFrEF and supraventricular arrhythmias. This regimen aims to suppress neurohormonal hyperactivation, promote reverse remodeling, and lower mortality. For HFpEF, pharmacotherapy focuses primarily on lowering elevated filling pressures and addressing comorbidities. SGLT2I represents the primary disease-modifying treatment with proven clinical benefit. Selected therapies such as ARNI and MRA offer modest benefits in subgroups [20]. In the acute phase, intravenous diuretics, vasodilators, inotropic agents, or morphine are used to relieve pulmonary congestion, hypoxemia, and anxiety, while chronic management focuses on oral medications that maintain fluid balance and slow disease progression [21]. Although these drugs cannot fully reverse structural damage, they transform HF into a clinically manageable chronic disease when combined with devices, lifestyle modification, and, in advanced cases, heart transplantation or mechanical circulatory support. Target-specific therapies are essential in HF because the disease is driven by multiple maladaptive pathways, including neurohormonal activation, remodeling, and inflammation [22,23,24]. Current guideline-directed treatment already relies on several drug classes acting on different mechanisms with improved outcomes but still leaves unmet needs for more precise interventions. Therefore, new targeted agents are needed to better control disease progression, reduce hospitalizations, and increase the likelihood of survival.

### 1.4. Role of FOXO3 in HF

Biomarkers, especially B-type natriuretic peptide (BNP), N-terminal pro-B-type natriuretic peptide (NT-proBNP), and high-sensitivity cardiac troponin (hs-cTn), are now central to defining HF, because they provide objective biochemical evidence of myocardial wall stress and ongoing injury that complement symptoms and imaging [25,26]. BNP and NT-proBNP play a central role in supporting the diagnosis, whereas hs-cTn reflects myocardial injury and is particularly valuable for prognostic assessment [1]. From a mechanistic standpoint, these biomarker trajectories may reflect upstream aging and stress pathways, including FOXO3 regulated oxidative stress, mitochondrial dysfunction, and cellular senescence, suggesting that future mechanism-based biomarkers could bridge canonical HF markers with FOXO3-driven cardioprotective signaling. FOXO3 is a forkhead transcription factor regulating oxidative stress responses, apoptosis, autophagy, and cardiac remodeling [27,28,29]. Although there is the substantial overlap among FOXO family members such as FOXO1, FOXO3, and FOXO4, FOXO3 is typically chosen as the focus in HF because it sits at the intersection of the HF-relevant processes such as oxidative stress sensing, mitochondrial quality control, autophagy, and stress-responsive transcription in cardiac tissue [30]. FOXO1 works primarily as a metabolic gatekeeper. In the heart, FOXO1 is heavily implicated in metabolic remodeling, endothelial inflammation, and post-ischemic remodeling, but its dominant effects are often metabolic rather than global stress integration. FOXO3 functions as the oxidative stress sensor with the anti-oxidant and autophagy gene repertoire, and control mitochondrial quality control, DNA repair, and apoptosis. It is positioned to coordinate survival or death decisions under chronic stress related to HF progression. FOXO4 is more specialized in p53-dependent senescence programs which is important in aging and some vascular contexts, but less central to cardiomyocyte-intrinsic stress integration than FOXO3. Notably, recent clinical studies have provided direct human evidence supporting a functional role for FOXO3 in HF pathophysiology and recovery. In patients with virus-negative inflammatory cardiomyopathy, carriers of the FOXO3 gain-of-function SNP rs12212067 exhibited significantly greater improvement in LVEF and clinical symptoms over 6 months, accompanied by reduced myocardial inflammatory infiltrates, suggesting that enhanced FOXO3 activity modulates cardiac immune responses and promotes clinical recovery [31]. Complementing these findings, a 2026 transcriptomic study of end-stage HF patients undergoing left ventricular assist device support identified FOXO3 among key genes associated with metabolic and inflammatory remodeling during mechanical unloading, demonstrating that elevated myocardial FOXO3 expression correlates with favorable molecular signatures of cardiac recovery [32]. Together, these human data substantiate the translational relevance of FOXO3 as a therapeutic target in HF, bridging mechanistic insights from preclinical models to clinical outcomes. FOXO3 in HF is context-dependent. It can be cardioprotective when it supports stress resistance and survival pathways, but it can also be harmful when overactivated and linked to apoptosis, inflammation, and maladaptive remodeling [33]. In the heart, FOXO3 activation can be protective by upregulating anti-oxidant genes such as manganese superoxide dismutase 2 (MnSOD2), catalase, and peroxiredoxin III [34]. For example, overexpression of FOXO3 in mouse hearts and H9c2 cells enhanced autophagic flux, suppressed ROS and mTOR, reduced fibrosis and ventricular remodeling, and improved cardiac function, suggesting that FOXO3 activation alleviates doxorubicin-induced HF [35]. Also, FOXO3 can promote cardiomyocyte survival by helping maintain calcium homeostasis and by inducing protective genes such as CITED2 and PINK1, which may reduce ischemia/reperfusion-related injury in the heart [36]. Shi et al. reported that homocysteine suppressed FOXO3 via Akt-mediated phosphorylation and nuclear exclusion in cardiac fibroblasts, promoting fibrosis. FOXO3 overexpression reversed these effects, highlighting Akt/FOXO3 as an anti-fibrotic target [37]. In contrast to the above studies, FOXO3 deficiency increased cardiomyocyte proliferation and improved adult heart function, emphasizing that timing and degree of FOXO3 activity are critical in HF [38]. A recent study notes that FOXO3 may mediate IL-18-induced cardiac inflammation and dysfunction through CXCL16 expression, which supports a detrimental role in HF-related contexts [39]. In these cases, genetic or pharmacologic inhibition of FOXO3 reduced injury, indicating that excessive FOXO3 activity can be harmful. In addition, in a HF transgenic mouse model, constitutively active FOXO3 overexpression induced significant cardiac atrophy and excessive autophagy through B-cell lymphoma 2/adenovirus E1B 19 kDa interacting protein 3 (BNIP3)-dependent mechanisms. This led to mitochondrial loss, sarcomere disruption, and rapid cardiac dysfunction, demonstrating that FOXO3 overactivation drives pathological myocardial atrophy [40]. Thus, activation of FOXO3 can promote cardiomyocyte proliferation and survival, potentially counteracting the detrimental effects of oxidative stress and inflammation. However, dysregulation of FOXO3 can also contribute to maladaptive responses in HF, necessitating careful consideration of its role in therapeutic strategies.

### 1.5. Outline of Review

FOXO3-targeting compounds are gaining significant attention in the field of pharmacology for their potential applications in treating various diseases, including HF. These compounds work by modulating the activity of FOXO3, a transcription factor crucial for regulating cellular processes such as autophagy, apoptosis, and oxidative stress resistance [27]. In this review, we structured narrative reviews of the growing importance of FOXO3-modulating compounds in HF treatment, focusing on findings published between 2006 and 2026. The primary aim of this review was to identify and introduce all reported modulators that can alter FOXO3 activity, regardless of whether the modulation was direct or indirect. Recent studies were identified through comprehensive searches on Google Scholar and PubMed using keywords such as phytochemicals and pharmaceuticals with HF and cardiac dysfunction. Covering diverse cellular and animal models across various therapeutic contexts, this article synthesizes cutting-edge insights and trends in FOXO3 modulation for HF management while detailing their underlying mechanisms. Ultimately, it seeks to guide future HF therapeutic development and serves as an essential reference for researchers and clinicians.

## 2. Phytochemicals Modulating FOXO3 in HF

### 2.1. Well-Studied Phytochemicals

Several recent papers link FOXO3 modulation by phytochemicals to improvement in HF symptoms. Resveratrol is the most extensively studied phytochemical that has demonstrated beneficial effects in improving HF. Resveratrol modulates FOXO3 in cardioprotective contexts through context-dependent activation or inhibition, primarily via SIRT1, SIRT3 or PI3K/Akt pathways, balancing nuclear and cytoplasmic localization to optimize anti-oxidant defense, apoptosis suppression, autophagy, and mitochondrial homeostasis. Activation of SIRT1 enhances FOXO3 transactivation of anti-oxidant genes such as SOD2, thereby alleviating oxidative stress and prolonging survival in HF models [41,42,43,44]. Additional mechanisms include miR-155 downregulation to suppress FOXO3-related networks in hypertrophy [45] and re-establishment of SIRT1/SIRT3-Mfn2-Parkin-PGC-1α axis for mitophagy [46]. Resveratrol alleviates cardiotoxicity via the SIRT3/FOXO3 axis and improves cardiac pathology and mitophagy disturbance in vitro and in vivo models through nuclear accumulation of FOXO3 [47,48,49]. Furthermore, the zebrafish model enabled clear visualization and validation of resveratrol’s cardioprotective effects through FOXO3 activation against oxidative damage and cell death [50]. In contrast to resveratrol’s canonical activation of FOXO3 via SIRT1 deacetylation for anti-oxidant defense in HF, two studies demonstrated the cardioprotection effect of resveratrol through FOXO3 inhibition in aging and diabetic contexts. Synergistic SIRT1 and PI3K/Akt stimulation by resveratrol promotes FOXO3 phosphorylation, enforcing cytoplasmic sequestration to block nuclear-driven apoptosis in senescent rat hearts [51]. Likewise, in diabetic cardiomyopathy, resveratrol restores Akt/FOXO3 phosphorylation, suppressing hyperglycemia-induced cardiomyocyte apoptosis and structural deterioration [52]. These findings reveal FOXO3’s context-dependent duality such as activation for adaptation and inhibition for survival as a nuanced therapeutic target. Thus, recent studies position FOXO3 as a central integrator of resveratrol’s multifaceted cardio protection, fine-tuning stress resistance, cell survival, and bioenergetic homeostasis in pathological contexts. Quercetin has been reported to modulate FOXO3 signaling in HF. In H9c2 rat cardiomyocytes, quercetin attenuated isoproterenol-induced cardiotoxicity through a FOXO3-mediated mechanism, indicating improved cell survival and reduced injury [53]. In a separate study, quercetin prevented isoprenaline-induced myocardial fibrosis by modulating the miR-223-3p/FOXO3 pathway and promoting autophagy, thereby suppressing pathological remodeling [54]. More recently, quercetin combined with exercise was shown to mitigate apoptosis and cardiomyopathy in diabetic obese rats via the PI3K/Akt/FOXO3 pathway by downregulating FOXO3 expression [55]. These findings support FOXO3 as a key molecular mediator of the cardioprotective actions of quercetin across different experimental models. Curcumin and its analogs exert cardioprotective effects by reducing oxidative stress, apoptosis, and pathological remodeling in diabetic or ischemic hearts. Curcumin analogue C66 was shown to improve diabetic cardiomyopathy in mice mainly through inhibition of JNK2 and activation of PI3K/Akt/FOXO3 signaling, which was associated with less cardiac inflammation, fibrosis, oxidative stress, and apoptosis [56]. Tetrahydro curcumin, another kind of curcumin analogues, ameliorated post-infarction cardiac dysfunction and remodeling by reducing oxidative stress, preserving mitochondrial function, and activating the SIRT3/FOXO3 pathway [57]. Curcumin also attenuated myocardial ischemia–reperfusion injury by suppressing autophagy-dependent ferroptosis through regulation of SIRT1 or the Akt/FOXO3 signaling axis [58]. Taken together, these findings suggest that curcumin and its analogues may contribute to cardioprotection through the modulation of multiple stress-response pathways, including JNK2, Akt, SIRT1, SIRT3, and FOXO3 signaling. Ginsenosides display cardioprotective effects through diverse signaling pathways, particularly by modulating JNK, Akt, SIRT1, SIRT3, and FOXO3. Ginsenoside Rb3 protects H9c2 rat cardiomyocytes against ischemia–reperfusion injury by inhibiting JNK-mediated NF-κB and the Akt/FOXO3 pathway, reducing inflammation and apoptosis [59]. Ginsenoside Rg1 was reported to regulate the Akt/FOXO3 signaling pathway, leading to downregulation of FOXO3 and alleviating sepsis-induced myocardial apoptotic damage in lipopolysaccharide (LPS)-induced sepsis mice [60]. Similarly, ginsenoside Rc ameliorates septic cardiomyopathy by downregulating FOXO3 expression and altering macrophage polarization to reduce cardiomyocyte damage in LPS-induced sepsis mice [61]. Ginsenoside Rh1 mitigates mitochondrial dysfunction during myocardial ischemia by activating the SIRT3/FOXO3 pathway, preserving mitochondrial function in permanent left anterior descending artery (LAD) ligation mice [62]. Taken together, these findings show that ginsenosides target oxidative stress, inflammation, apoptosis, and mitochondrial dysfunction to protect the heart from ischemia, sepsis, and other cardiac injuries. The common theme across these studies is the regulation of FOXO3, which is central to cardiac stress responses and cell survival. Ginsenosides have shown potential in the context of inflammatory heart diseases.

As shown in Table 1, phytochemicals including resveratrol, quercetin, curcumin, and ginsenosides converge on FOXO3 as a central hub mediating cardioprotection in both cell and animal models of HF through context-dependent modulation. In cell-based experiments, resveratrol was tested in endothelial cells, cardiomyocytes, and primary cardiac cells at concentrations ranging from 10 to 80 μM for 24 to 48 h. In animal studies, resveratrol was administered to models such as TO-2 cardiomyopathic hamsters, rats, and dystrophin-deficient Mdx mice at doses of 4 to 400 mg/kg for 4 to 120 weeks. Resveratrol exhibits duality activating FOXO3 via SIRT1 or SIRT3 for anti-oxidant defense and autophagy in canonical HF but inhibiting it through PI3K/Akt-mediated phosphorylation to block nuclear-driven apoptosis in aging and diabetic contexts. Quercetin and curcumin analogs reinforce FOXO3 signaling via SIRT1, SIRT3, PI3K/Akt, and JNK2 pathways to suppress oxidative stress and apoptosis, while ginsenosides Rb3, Rg1, Rc, and Rh1 regulate FOXO3 alongside JNK, Akt, SIRT1, and SIRT3 to mitigate inflammation, apoptosis, and mitochondrial dysfunction in ischemia and sepsis. These phytochemicals fine-tune stress resistance, cell survival, and bioenergetic homeostasis, positioning FOXO3 as a nuanced therapeutic target that integrates diverse signaling networks across pathological contexts. Targeting FOXO3 with this phytochemical repertoire thus offers multifaceted, adaptable strategies for treating HF and cardiac injuries driven by oxidative stress, inflammation, and metabolic dysfunction.

### 2.2. Emerging Phytochemicals

In addition to resveratrol, quercetin, curcumin, and ginsenosides, several other phytochemicals have been reported to ameliorate HF symptoms through FOXO3 modulation mechanisms including SIRT1, SIRT3, Akt, Erk, or AMPK/FOXO3 signaling pathway in various HF model systems. Echinacoside, mangiferin, and bergenin were associated with upregulation of SIRT1 together with increased FOXO3 and MnSOD2 expression, indicating activation of a sirtuin-mediated anti-oxidant defense program [64,65,66]. Interestingly, 5-demethyl nobiletin attenuated apoptosis in isoprenaline-induced atrial fibrillation mice by upregulating SIRT1 expression but downregulating phosphorylated FOXO3 expression [67]. In addition, acetovanillone enhanced the cardioprotective effect against cadmium-induced cardiac injury rat model by reducing necroptosis and oxidative stress through upregulation of SIRT1 expression, downregulation of FOXO3 expression, and increased MnSOD2 activity [68]. In doxorubicin-induced HF mice, daidzein attenuated cardiac dysfunction by modulating the SIRT3/FOXO3/MnSOD2 signaling pathway [69]. n-Tyrosol and puerarin modulated Akt-related signaling and reduced apoptosis, suggesting that suppression of Akt-driven pro-survival imbalance may contribute to FOXO3-dependent cardioprotection [70,71]. Oleanolic acid were linked to AMPK activation and increased FOXO3 expression, supporting a role for energy-sensing pathways in stress adaptation and redox homeostasis [72]. Interestingly, in diabetic Goto–Kakizaki rats, epigallocatechin-3-gallate alleviated myocardial mitochondrial dysfunction and autophagy by reducing the expression levels of AMPK, FOXO3, and MnSOD2 proteins [73]. In addition, herbacetin decreased pErk1/2 expression while increasing FOXO3 expression, implying that Erk inhibition may further facilitate FOXO3-mediated cardioprotective effects [74].

As shown in Table 2, phytochemicals including n-tyrosol, oleanolic acid, epigallocatechin gallate, puerarin, echinacoside, mangiferin, bergenin, daidzein, acetovanillone, 5-demethyl nobiletin, and herbacetin, converge on FOXO3 as a central regulatory node mediating cardioprotection in HF cellular and animal models. In cell-based systems, these compounds were tested primarily in cardiomyocytes and cardiac fibroblasts at micromolar concentrations over 18 to 24 h, whereas in animal studies they were administered to models of myocardial infarction, pressure overload, atrial fibrillation, doxorubicin-induced HF, diabetic HF, and cadmium-induced HF at doses ranging from 5 to 100 mg/kg for periods of 6 days to 12 weeks. Overall, these findings indicate that several kinds of phytochemical regulation of FOXO3 are coordinated through SIRT1, SIRT3, Akt, Erk, or AMPK signaling pathways to suppress apoptosis, attenuate oxidative stress, and improve cardiac cell survival. Notably, several compounds enhance FOXO3-dependent anti-oxidant defenses, while n-tyrosol, epigallocatechin gallate, and acetovanillone suppress FOXO3 expression or modulate its nuclear activity to preserve cell survival. These findings raise the possibility that phytochemical modulation of FOXO3 may contribute to the context-dependent regulation of HF-related processes.

### 2.3. Plant Extracts

Several plant-derived extracts have been reported to confer cardioprotection in HF-related experimental models through modulation of the FOXO3 axis and its upstream pathways such as Akt and AMPK in various HF models. White wine, Sanwei-Tanxiang formulation, blueberry extract, and Qili qiangxin formulation were shown to reduce apoptosis, accompanied by increased phosphorylated Akt and phosphorylated FOXO3 expression or decreased FOXO3 expression [75,76,77,78]. However, Gentianella acuta extract demonstrated beneficial effects in the TAC-induced HF rat model, by suppressing autophagy and decreasing phosphorylated Akt and phosphorylated FOXO3 expression [79]. Interestingly, Linggui Zhugan formulation alleviates doxorubicin-induced cardiotoxicity by reduction in apoptosis and inhibition of oxidative damage, with AMPK-driven FOXO3 phosphorylation as a key mechanism [80]. Panax notoginseng saponins and Polygonum cuspidatum extract attenuated cardiac injury in old rats or Angiotensin II-induced HF mice, with activation of FOXO3-associated SOD expression [81,82]. Collectively, these findings suggest that herbal extracts containing diverse phytochemicals may influence FOXO3-centered signaling networks, potentially contributing to cellular survival, reduced oxidative stress, and the preservation of cardiac function in HF.

As shown in Table 3, plant extracts including white wine, Panax notoginseng, Sanwei-Tanxiang, blueberry, Gentianella acuta, Polygonum cuspidatum, Linggui Zhugan, and Qili qiangxin converge on FOXO3 as a key regulatory node in HF models. In H9c2 rat cardiomyocytes, these extracts were mainly tested from 50 μg/mL to 10 mg/mL concentrations for 24 h, whereas in animal studies they were administered to LAD ligation, TAC-induced, angiotensin II-induced, and doxorubicin-induced HF models at doses ranging from 60 mg/kg to 20 g/kg or 6.5 mL/kg over 1 week to 30 days. Overall, these findings suggest that FOXO3-mediated cardioprotection is regulated through Akt or AMPK and related oxidative stress and apoptosis pathways. Notably, there was no study using plant extracts in those papers that specifically examined the relationship between SIRT1 or SIRT3 and FOXO3. Although many phytochemicals and plant extracts show promising cardioprotective effects in cellular and animal studies, robust clinical evidence supporting their efficacy, safety, and optimal dosing remains insufficient. The clinical translation of phytochemicals remains limited by their generally poor oral bioavailability, extensive metabolism, and rapid systemic elimination. Therefore, well-designed pharmacokinetic studies and randomized clinical trials are required before their therapeutic potential can be reliably translated into clinical practice.

## 3. Pharmaceuticals Modulating FOXO3 in HF

Several studies have also investigated pharmaceuticals that regulate FOXO3 in HF models. Statins, a class of cholesterol-lowering drugs, exhibit complex FOXO3-related effects in cardiac cell and animal models. A study revealed that p21CIP1/WAF1 acts as a downstream target of FOXO3 to mediate lovastatin-induced anti-hypertrophic effects through inhibition of Rho-kinases in rat primary cardiomyocytes [83]. However, other findings show that simvastatin induces mitochondrial dysfunction and apoptosis through increase in atrogin-1 expression and translocation of FOXO3 in H9c2 cardiomyocytes and normal mice, suggesting potential adverse effects of FOXO3 in the cardiac system [84]. In another study, simvastatin promotes cardiac microvascular endothelial cells proliferation, migration, and survival by phosphorylation of Akt, p70S6K, and FOXO3, demonstrating cell-type-specific beneficial effects of FOXO3 in the cardiac system [85]. As another pharmaceutical regulating FOXO3 in the cardiac system, dapagliflozin, a kind of sodium-glucose cotransporter 2 inhibitor, promotes metabolic reprogramming against myocardial infarction by activation of phosphorylation of JNK and reduction in FOXO3 expression, thereby enhancing cardiomyocyte survival and suppressing apoptosis in H9c2 cardiomyocytes and LAD ligation rats [86]. Additionally, dapagliflozin ameliorates atrial fibrillation in streptozotocin-induced diabetes mice and high-glucose-treated HL-1 cells by increasing SIRT1 and phosphorylated FOXO3 expression to reduce apoptosis, restore autophagy and mitophagy, and improve calcium channel activity, thereby mitigating atrial fibrosis [87]. Metoprolol, a kind of β-blocker, increases the expression of β3-adrenoceptors in the diabetic heart of streptozotocin-induced diabetes mice, with effects on regulation of the Akt/FOXO3 signaling pathway [88]. Entinostat, a kind of class I histone deacetylase inhibitor, exerts cardiac protection from ischemia–reperfusion through upregulation of nuclear FOXO3, MnSOD2, and catalase expression in ex vivo heart from Sprague Dawley rats [89]. Artesunate, an anti-malarial drug, alleviates HF by suppressing apoptosis, inhibiting ROS production, and ameliorating mitochondrial damage through the SIRT1/FOXO3/MnSOD2 pathway in doxorubicin-induced injured H9c2 cardiomyocytes [90]. Lastly, dronedarone, a kind of multi-channel blocker, attenuates myocardial hypertrophy through regulating the SIRT1/FOXO3 signaling pathway in H9c2 cardiomyocytes and TAC-induced HF rats [91]. In addition, the study demonstrates that dronedarone reduces cell size and suppresses hypertrophy-associated genes including atrial natriuretic peptide, B-type natriuretic peptide, and β-MHC in angiotensin II-induced hypertrophy H9c2 cells.

As shown in Table 4, the pharmaceuticals such as lovastatin, simvastatin, dapagliflozin, metoprolol, entinostat, artesunate, and dronedarone plays a role in FOXO3 expression as a key regulatory node in HF in vitro and in vivo models. In cellular experiments, these pharmaceuticals were tested in H9c2 rat cardiomyocytes, HL-1 mouse cardiomyocytes, or cardiac endothelial cells at concentrations ranging from 1 to 20 μM for 24 to 48 h, whereas in animal studies they were administered in C57BL/6 mice, LAD ligation rats, streptozotocin-induced diabetic mice, ex vivo heart preparation, and TAC-induced HF rats at doses ranging from 10 to 90 mg/kg over periods of 1 to 8 weeks. These studies suggest that cardioprotection effect of the above pharmaceuticals is related to reduction in oxidative stress, apoptosis, and mitochondrial function via regulation of the Akt, AMPK, JNK, and SIRT1 signaling pathway. Notably, a study suggests that simvastatin may induce mitochondrial toxicity in HF models, potentially through FOXO3 activation, highlighting the need for caution in its clinical use [84]. Consistent with findings from other studies on phytochemicals and natural extracts, research involving pharmacological agents further suggests that cardioprotective effects may be associated with the activation or inhibition of FOXO3, highlighting the potential importance of context-dependent regulation of FOXO3 activity for therapeutic benefit.

## 4. Other Chemicals Modulating FOXO3 in HF

Besides phytochemicals and pharmaceuticals, there are also studies on other compounds that have been shown to exert cardioprotective effects in experimental HF models by modulating the FOXO3 signaling axis. Enzymatic and peptide-based interventions have demonstrated cardioprotective effects in HF and related cardiovascular injury models. Bromelain, proteolytic enzymes from pineapple, attenuate ischemia–reperfusion injury ex vivo heart model from rats by activating Akt and increasing FOXO3 phosphorylation, which in turn inhibits FOXO3 transcriptional activity and reduces apoptosis and infarct size [92]. In contrast, two bioactive peptides from potato (N-DIKTNKPVIF-C) and soybean (N-VHVV-C) elevate FOXO3 expression and simultaneously activate the AMPK/SIRT1/PGC-1α/FOXO3 axis to limit hypertrophy and fibrosis in spontaneously hypertensive rat model [93,94]. These peptides, especially when combined with exercise, upregulate AMPK, SIRT1, PGC1α, and FOXO3, promoting cell survival and mitochondrial biogenesis in pressure-overloaded hearts. Elabela, a peptide hormone, has been reported to regulate SIRT3-mediated inhibition of oxidative stress in streptozotocin-induced diabetes mice through FOXO3 deacetylation, further preventing injury in a FOXO3-dependent manner [95]. Thus, bromelain suppresses FOXO3-mediated proapoptotic signaling via post-translational inhibition, whereas two bioactive peptides, and elabela enhance FOXO3 activity through increased expression or deacetylation-linked SIRT pathway activation. Together, these findings indicate that both inhibition and activation of FOXO3-dependent pathways can yield cardioprotection, depending on the context of the disease and molecular mechanism in HF and diabetic heart injury. In addition, taurine, a kind of non-standard amino acid, reduced infarct size and improved cardiac function in a rat model of myocardial infarction induced by LAD ligation [96]. The cardioprotective effects were enlaced with exercise training and mediated through decreased expression of Akt and increased expression of FOXO3. Recent studies highlight that ketone body metabolites can exert cardioprotective effects by modulating FOXO3-related pathways. The ketone body β-hydroxybutyrate prevents myocardial oxidative stress in LPS-induced sepsis mice [97]. Administration of β-hydroxybutyrate–1,3-butanediol monoester increases circulating β-hydroxybutyrate levels in LSP-induced septic mice. Additionally, β-hydroxybutyrate treatment upregulates the expression of FOXO3 and MnSOD2 in H9c2 cells. Similarly, 1,3-butanediol enhances autophagy and reduces apoptosis via the PI3K/Akt/FOXO3 pathway, thereby attenuating cardiac remodeling after myocardial infarction and improving post-injury structural and functional outcomes in LAD ligation rats [98]. Together, these findings indicate that ketone body metabolites, whether delivered as precursors (1,3-butanediol) or as free ketones (β-hydroxybutyrate), can protect the heart by engaging FOXO3 to regulate autophagy, oxidative stress responses, and remodeling. These studies position ketone metabolism as a promising metabolic axis for therapeutic intervention in HF. Modulation by upstream regulatory chemicals influences the dual role of FOXO3 in cardiomyocyte stress responses, depending on the signaling context. In H9c2 cardiomyocytes exposed to doxorubicin, hydrogen sulfide, an endogenous gaseous signaling molecule, activates the PI3K/Akt pathway, increases phosphorylation of FOXO3, and suppresses its nuclear translocation, thereby attenuating apoptosis and cytotoxicity [99]. Furthermore, lithium chloride, a kind of pharmacological inhibitor of GSK3β, suppresses FOXO3 activity and protects the heart from inflammation-mediated damage in LPS-induced sepsis rats [100]. In contrast, during pathological heart hypertrophy, JNK modulates FOXO3 to upregulate BNIP3, a mitochondrial death and mitophagy marker, promoting mitochondrial dysfunction and cell death, and this mechanism has been supported by experiments using 3-methyladenine, a widely used pharmacological inhibitor of autophagy [101].

Table 5 summarizes several agents, including bromelain, 3-methyadenine, hydrogen sulfide, lithium chloride, elabela, β-hydroxybutyrate, VHVV peptide, DKTNKPVIF peptide, taurine, and 1,3-butanediol, all of which were reported to influence FOXO3-related signaling in HF-relevant experimental systems. In vitro, these compounds were examined mainly in primary rat cardiomyocytes or H9c2 cells under relatively short exposures, whereas in vivo studies used ex vivo ischemia/reperfusion hearts, aortic banding rats, LPS-induced sepsis models, streptozotocin-induced diabetic mice, spontaneously hypertensive rats, and LAD ligation rats, with treatment durations ranging from days to several weeks. Across these models, the reported effects were largely associated with reduced apoptosis and attenuation of oxidative injury, together with changes in upper signaling pathways involving Akt, JNK, SIRT1, GSK3β, and AMPK. Notably, some interventions such as bromelain, hydrogen sulfide, and lithium chloride downregulated FOXO3 expression leading to reduction in apoptosis, suggesting that FOXO3 regulation is not uniformly protective or harmful but instead depends on the specific compound, model, and signaling environment.

## 5. Limitations and Future Directions

FOXO3-targeting approaches have shown promising cardioprotective effects in various HF models. Nevertheless, their development as therapeutic strategies or functional interventions for HF management faces several important limitations, including context-dependent signaling outcomes, cell-type specificity, and the risk of maladaptive effects due to the pleiotropic nature of FOXO3 regulation. Firstly, FOXO3 exerts dual roles in HF, acting as a cardioprotective factor under certain stress conditions but contributing to cardiomyocyte injury when dysregulated. Most cardiac studies emphasize FOXO3 activation or genetic suppression, highlighting its context-dependent role as either protective or maladaptive in heart disease [30,102,103]. For example, cardiomyocyte-specific FOXO3 knockout enlarges infarct size after I/R and reduces anti-oxidant and autophagy-related gene expression, supporting a protective role [27,36], whereas activation of the FOXO3–BNIP3 axis in an HFpEF model promotes mitochondrial Ca2+ overload, fragmentation, and apoptosis in a maladaptive manner [104,105]. In addition, recent studies support the view that FOXO3 is a context-dependent regulator of cardiac remodeling or cell fate. FOXO3 has been shown to blunt pathological cardiac hypertrophy and induction of anti-oxidant programs, indicating that it can counter maladaptive growth responses in stressed cardiomyocytes [106]. At the same time, excessive FOXO3 activation has been associated with cardiomyocyte atrophy, autophagy, reduced cardiac mass, and impaired performance, suggesting that unrestrained FOXO3 signaling may become maladaptive [40]. The effects of FOXO3 should be interpreted in a context-dependent manner. In addition, the apparent discrepancy among studies using the same chemicals may reflect differences in disease model, stress intensity and duration, cardiac phenotype, cell type, and the magnitude or timing of FOXO3 modulation. Its therapeutic effects depend on achieving the appropriate level and timing of pathway modulation. Thus, it is necessary to quantify the pattern whereby FOXO3 is protective under early/low-intensity stress but harmful under chronic/high-intensity stress and to define molecular signatures for intervention at each phase. More specifically, FOXO3 activity (nuclear/cytoplasmic localization and phosphorylation/acetylation status) and target gene patterns (anti-oxidant, autophagy, and apoptosis) should be profiled under specific compound treatment over various time points in HF models. Secondly, most studies focus on upstream modulators such as SIRT1/3, Akt and AMPK that promote FOXO3 post-translational modification and regulate nuclear/cytosol localization, which indirectly governs FOXO3 transcriptional activity in experimental HF models [107,108,109,110]. A study showed that SIRT3 deacetylates and activates FOXO3 in cardiomyocytes, upregulating MnSOD2 and catalase to lower ROS and thereby suppress cardiac hypertrophy and fibrosis in mice [111]. Also, Akt phosphorylates FOXO3 and promotes its nuclear exclusion in rat ventricular myocytes, which is associated with cardiomyocyte hypertrophy and size [112]. One key limitation is that indirectly targeting FOXO3 via upstream kinases/deacetylases produces pleiotropic effects, so the net impact on FOXO3 transcriptional output in the heart can be unpredictable and may not consistently improve cardiac function. A more direct and target-specific strategy is needed to precisely modulate FOXO3-dependent transcriptional programs in HF. This approach could identify compounds that interact with FOXO3 itself. For example, small-molecule binders, DNA-binding-domain inhibitors, or PROTAC-based degraders can selectively alter FOXO3 activity in a context-dependent manner [113,114,115,116]. In addition, a recent study reports that a small synthetic molecule designed to target the forkhead DNA-binding domain on FOXO3 can selectively turn off transcription of pro-apoptotic genes such as Bim or NOXA while preserving beneficial effects such as reducing ROS [113]. Thus, future work should focus on direct FOXO3 targeting approach that could be leveraged to uncouple maladaptive function from beneficial activity via emerging chemical tools, thereby refining the therapeutic window of FOXO3 modulation in experimental HF models. Thirdly, FOXO3 is broadly expressed across multiple tissues, and its signaling network is not limited to the heart [102,117,118,119]. This widespread distribution raises an important challenge for therapeutic development, as systemic FOXO3 modulation may produce off-target effects in non-cardiac organs. Therefore, achieving tissue specificity is essential for translating FOXO3-targeted strategies into clinically useful HF therapies [120,121,122]. A cardiac-selective drug delivery system would help preserve the beneficial effects of FOXO3 in cardiomyocytes while minimizing unwanted effects in other tissues [123,124]. This could be accomplished through targeted nanoparticles, viral vectors with cardiac tropism, or prodrug strategies activated specifically in the heart. Alternatively, compounds designed to act only under disease-specific conditions may improve selectivity and reduce systemic toxicity. Such approaches are particularly important given the context-dependent and dual roles of FOXO3 in stress adaptation and injury. Without tissue-specific control, FOXO3 directed therapy may fail to distinguish between protective and maladaptive signaling. Accordingly, future studies should emphasize delivery platforms and molecular designs that confine FOXO3 modulation and this would improve both the safety and therapeutic precision of FOXO3-based intervention in HF. Fourthly, FOXO3 has emerged as an important context-dependent regulator of cardiac homeostasis, but its therapeutic modulation may require distinct strategies depending on the disease stage. In the setting of preventive cardiovascular health, FOXO3 activation may be advantageous because it supports cellular stress resistance, anti-oxidant defense, autophagy, and metabolic resilience, thereby helping maintain myocardial integrity before injury develops. Consistent with this view, previous studies have suggested that FOXO3 contributes to cardiomyocyte survival under oxidative stress and that FOXO3-related signaling may be linked to longevity and protection against age-associated cardiovascular dysfunction [36,102,125]. By contrast, in acute cardiac disease states, such as ischemia–reperfusion injury, chemotherapy-induced cardiomyopathy, or inflammatory cardiac injury, FOXO3-directed intervention should be designed according to the main pathological mechanism. In these settings, transient enhancement of FOXO3 activity may be beneficial when the primary problem is oxidative injury, mitochondrial dysfunction, or impaired autophagic flux, whereas suppression of maladaptive FOXO3 signaling may be preferable when FOXO3 contributes to excessive atrophy, cardiac remodeling, or pathological immune activation [34,40,126,127,128]. Therefore, FOXO3 should not be viewed as a uniformly beneficial or deleterious target, but rather as a nodal regulator whose therapeutic direction must be matched to the biological goal. For preventive applications, nutraceuticals or low-intensity pharmacological agents may be most appropriate for sustaining FOXO3-mediated cardioprotective programs over time. In acute therapeutic settings, more precise and time-restricted modulation will be required to maximize efficacy while minimizing the risk of disrupting adaptive cardiac responses. Fifthly, while a lot of preclinical evidence underscores FOXO3 as a compelling therapeutic candidate in HF models, several key hurdles continue to impede its clinical translation. The persistent gap between robust preclinical success and clinical translation for FOXO3-targeted HF therapies highlights critical limitations in translational trial design and patient classification [129,130,131]. A primary obstacle is the lack of validated, non-invasive circulating or imaging biomarkers capable of monitoring real-time FOXO3 activity in human cardiac tissue. Without accessible pharmacodynamic markers, evaluating target engagement or establishing optimal dosing protocols in clinical trials remains exceptionally challenging. To address the lack of non-invasive biomarkers for real-time FOXO3 activity in human cardiac tissue, several complementary strategies can be pursued, which include circulating miRNA linked to cardiac FOXO3 or extracellular vesicle based cardiac FOXO3 [132,133,134]. Additionally, preclinical studies predominantly utilize homogeneous, young animal models subjected to acute surgical interventions, which do not reflect the heterogeneous, multi-factorial etiology of human HF. Human HF populations exhibit vast genetic variability, age-related epigenetic changes, and diverse pharmacological backgrounds that can alter upstream FOXO3 regulatory networks. To overcome these translational bottlenecks, future research must emphasize identifying reliable surrogate biomarkers of cardiac FOXO3 activity, by which clinically aligned frameworks can be effectively designed for the translational FOXO3-targeted therapeutics.

## 6. Conclusions

**Figure 1:**
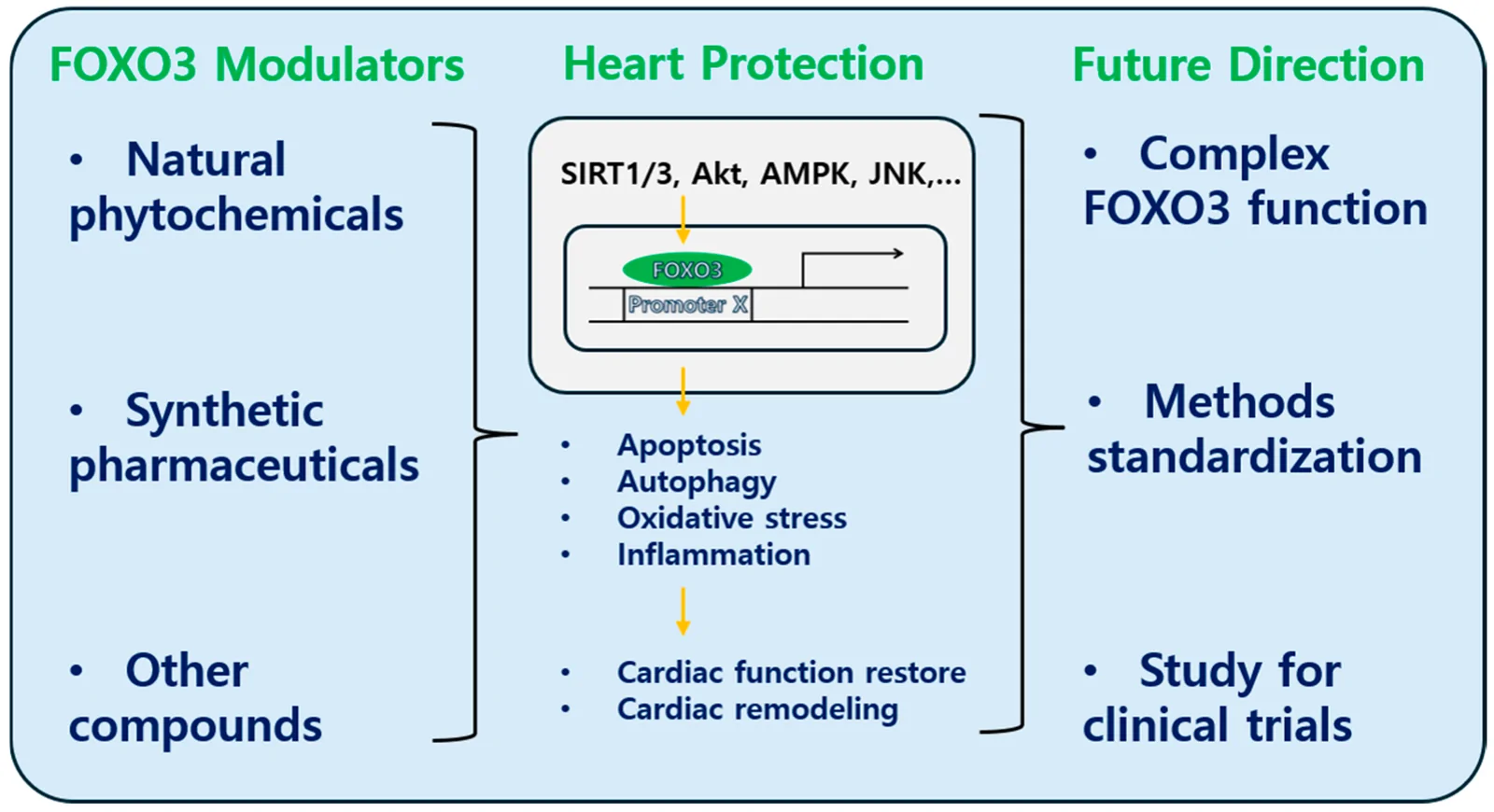
A comprehensive mechanistic figure summarizing the main concept of the review.

Over the past two decades (2006–2026), targeting the transcription factor FOXO3 has emerged as a potentially important therapeutic strategy for HF. As highlighted in this review, FOXO3 plays a central role in coordinating intracellular responses to oxidative stress, apoptosis, autophagy, and inflammation, thereby influencing cardiac cell survival and function (Figure 1). A wide range of compounds, including natural phytochemicals and synthetic pharmaceuticals, has demonstrated preclinical efficacy in improving cardiac function and attenuating pathological remodeling across diverse experimental models. However, the current evidence is derived predominantly from cellular and animal studies and therefore does not support strong claims regarding the therapeutic potential of FOXO3-targeting compounds in patients with HF. Clinical studies directly evaluating FOXO3 modulation, its downstream targets, or the FOXO3 dependence of drug-induced cardioprotection remain lacking. Substantial challenges must therefore be addressed before these experimental findings can be translated into clinical applications, including the context-dependent nature of FOXO3 signaling, methodological heterogeneity among HF models, uncertainty regarding the optimal direction and timing of FOXO3 modulation, and the pharmacokinetic and safety limitations of candidate compounds. In conclusion, research on FOXO3-targeting compounds highlights a promising yet largely preclinical therapeutic avenue for HF and underscores the complexity of developing effective and clinically validated pharmacotherapies.

## Figures and Tables

**Table 1:** Well-studied phytochemicals modulating FOXO3 in HF.

No.	Phytochemicals	Cell Model	Dose, Time	Animal Model	Dose, Time	Mechanism	Year, Ref.
1	Resveratrol	N/A	N/A	TO-2 cardiomyopathic hamsters	4 g/kg, 35 weeks	Increase in SIRT1, FOXO3, and MnSOD2 expression	2008, [41]
2	Resveratrol	N/A	N/A	Normal Sprague–Dawley rats	10 mg/kg, 120 weeks	Induction of autophagy Induction of nuclear translocation of FOXO3 Increase in SIRT1 and FOXO3 expression	2010, [47]
3	Resveratrol	EA.hy 926 human endothelial cells	30 μM, 24 h	N/A	N/A	Increase in SIRT1, FOXO3, and eNOS expression	2013, [42]
4	Resveratrol *	N/A	N/A	18-month-old rats	15 mg/kg, 4 weeks	Reduction in apoptosis Increase in SIRT1 and pFOXO3 expression	2014, [51]
5	Resveratrol	N/A	N/A	Dystrophin-deficient Mdx mice	400 mg/kg, 62 weeks	Induction of autophagy Induction of nuclear translocation of FOXO3 Increase in catalase and Cu/ZnSOD1 expression	2014, [43]
6	Resveratrol	H9c2 rat cardiomyocytes	80 μM, 24 h	Triptolide-induced cardiomyopathic mice	50 mg/kg, 24 h	Reduction in apoptosis Induction of nuclear translocation of FOXO3 Increase in SIRT3 and pFOXO3 expression	2016, [48]
7	Resveratrol	Mouse primary cardiomyocytes	10 μM, 24 h	Transverse aortic constriction-induced mice	150 mg/kg, 4 weeks	Increase in BRCA1 and FOXO3 expression	2016, [45]
8	Resveratrol *	Rat primary ventricular myocytes	10 μM, 24 h	Streptozotocin-induced diabetes rats	50 mg/kg, 8 weeks	Reduction in apoptosis Increase in pAkt and pFOXO3 expression Suppression of nuclear translocation of FOXO3	2017, [52]
9	Resveratrol	H9c2 rat cardiomyocytes	30 μM, 24 h	Dystrophin-deficient Mdx mice	50 mg/kg, 62 weeks	Induction of autophagy Induction of nuclear translocation of FOXO3 Increases in SIRT1 activity	2018, [49]
10	Resveratrol	Rat primary cardiomyocytes	20 μM, 24 h	N/A	N/A	Induction of autophagy Increase in SIRT1 and FOXO3 expression	2022, [46]
11	Resveratrol	AC16 human cardiomyocytes	40 μM, 24 h	Verapamil-induced HF zebrafish	40 μM, 72 h	Reduction in apoptosis Increase in SIRT1 and FOXO3 expression	2024, [63]
12	Resveratrol	N/A	N/A	High-fat and fructose-induced diabetes mice	45.5 mg/kg, 2 weeks	Inhibition of oxidative damage Increase in SIRT3, FOXO3, and catalase expression	2024, [44]
13	Quercetin	H9c2 rat cardiomyocytes	5 μM, 24 h	N/A	N/A	Increases in catalase activity Increase in FOXO3 expression	2017, [53]
14	Quercetin	Rat primary cardiac fibroblasts	50 μM, 48 h	Isoprenaline-induced atrial fibrillation rats	25 mg/kg, 3 weeks	Induction of autophagy Increase in FOXO3 expression	2021, [54]
15	Quercetin *	N/A	N/A	High fat and streptozotocin-induced diabetes rats	15 mg/kg, 8 weeks	Reduction in apoptosis Increase in PI3K and Akt expression Decrease in FOXO3 expression	2024, [55]
16	Monocarbonyl curcumin *	N/A	N/A	Streptozotocin-induced diabetes mice	5 mg/kg, 54 weeks	Reduction in apoptosis Increase in pAkt and pFOXO3 expression	2018, [56]
17	Tetrahydro curcumin	Mouse primary cardiomyocytes	3 μM, 3 h	LAD ligation mice	120 mg/kg, 4 weeks	Reduction in apoptosis Inhibition of oxidative damage Decrease in AcFOXO3 and AcSOD2 expression	2023, [57]
18	Curcumin *	H9c2 rat cardiomyocytes	10 μM, 48 h	LAD ligation rats	50 mg/kg, 4 weeks	Reduction in apoptosis, autophagy, and ferroptosis Increase in SIRT1, pAkt, and pFOXO3 expression Suppression of nuclear translocation of FOXO3	2025, [58]
19	Ginsenoside Rb3	H9c2 rat cardiomyocytes	5 μM, 24 h	N/A	N/A	Reduction in apoptosis Inhibition of oxidative damage Decrease in pAkt and pFOXO3 expression	2014, [59]
20	Ginsenoside Rg1 *	H9c2 rat cardiomyocytes	25 mM, 6 h	Lipopolysaccharide-induced sepsis mice	70 mg/kg, 6 weeks	Reduction in apoptosis and inflammation Increase in pAkt expression Decrease in FOXO3 expression	2024, [60]
21	Ginsenoside Rc *	H9c2 rat cardiomyocytes	10 µM, 48 h	Lipopolysaccharide-induced sepsis mice	20 mg/kg, 72 h	Reduction in apoptosis Inhibition of oxidative damage Increase in SIRT1 expression Decrease in FOXO3 expression	2025, [61]
22	Ginsenoside Rh1	H9c2 rat cardiomyocytes	25 µM, 12 h	LAD ligation mice	10 mg/kg, 1 weeks	Reduction in apoptosis Induction of mitophagy Inhibition of oxidative damage Increase in SIRT3 and FOXO3 expression Decrease in AcFOXO3 expression Induction of nuclear translocation of FOXO3	2025, [62]

N/A: Not available. pFOXO3: Phosphorylated forkhead box O3. AcFOXO3: Acetylated forkhead box O3. pAkt: Phosphorylated protein kinase B. MnSOD2: Manganese-dependent superoxide dismutase 2. Cu/ZnSOD1: Copper/Zinc Superoxide Dismutase 1. AcSOD2: Acetylated superoxide dismutase 2. eNOS: Endothelial nitric oxide synthase. HF: heart failure. MDX: Muscular dystrophy X-linked. LAD: Left Anterior descending artery. * means the suppression of FOXO3 for cardiac protection.

**Table 2:** Emerging phytochemicals modulating FOXO3 in HF.

No.	Phytochemicals	Cell Model	Dose, Time	Animal Model	Dose, Time	Mechanism	Year, Ref.
1	n-Tyrosol *	N/A	N/A	LAD ligation rats	5 mg/kg, 4 weeks	Reduction in apoptosis Increase in SIRT1, pAkt, and pFOXO3 expression	2008, [70]
2	Oleanolic acid	H9c2 rat cardiomyocytes	100 μM, 20 min	N/A	N/A	Reduction in mitochondrial membrane potential Increase in pAMPK and pFOXO3 expression	2009, [72]
3	Epigallocatechin gallate *	N/A	N/A	Diabetic Goto Kakizaki rats	100 mg/kg, 12 weeks	Reduction in autophagy Decrease in pAMPK, FOXO3, and MnSOD2 expression	2014, [73]
4	Puerarin	H9c2 rat cardiomyocytes	10 μM, 24 h	Aortic Banding mice	65 mg/kg, 7 weeks	Reduction in apoptosis Decrease in pAkt and pFOXO3 expression	2014, [71]
5	Echinacoside	AC16 human cardiomyocytes	50 μM, 24 h	Isoprenaline-induced atrial fibrillation rats	20 mg/kg, 2 weeks	Reduction in apoptosis Inhibition of oxidative damage Increase in SIRT1, FOXO3, MnSOD2 expression	2021, [64]
6	Mangiferin	H9c2 rat cardiomyocytes	20 μM, N/A	LAD ligation mice	40 mg/kg, 2 weeks	Reduction in apoptosis Increase in SIRT1 and AcFOXO3 expression	2021, [65]
7	Bergenin	H9c2 rat cardiomyocytes	30 μM, 18 h	N/A	N/A	Reduction in apoptosis Inhibition of oxidative damage Increase in SIRT1, FOXO3, MnSOD2 expression	2022, [66]
8	Daidzein	H9c2 rat cardiomyocytes	1 μM, N/A	Doxorubicin-induced HF mice	10 mg/kg, 2 weeks	Reduction in apoptosis Inhibition of oxidative damage Increase in SIRT3, FOXO3, MnSOD2 expression	2022, [69]
9	Acetovanillone *	N/A	N/A	Cadmium-induced HF rats	25 mg/kg, 10 days	Reduction in necroptosis Inhibition of oxidative damage Increase in SIRT1 expression Decrease in FOXO3 expression Increases in MnSOD2 activity	2023, [68]
10	5-Demethyl nobiletin	Rat primary cardiac fibroblasts	20 μM, 24 h	Isoprenaline-induced atrial fibrillation mice	30 mg/kg, 3 weeks	Reduction in apoptosis Increase in SIRT1 expression Decrease in pFOXO3 expression	2024, [67]
11	Herbacetin	H9c2 rat cardiomyocytes	50 μM, 24 h	Doxorubicin-induced HF mice	20 mg/kg, 6 days	Reduction in apoptosis Inhibition of oxidative damage Decrease in pErk expression Increase in FOXO3 expression	2026, [74]

N/A: Not available. pFOXO3: Phosphorylated forkhead box O3. AcFOXO3: Acetylated forkhead box O3. pAkt Phosphorylated protein kinase B. pAMPK: Phosphorylated AMP-activated protein kinase. pErk: Phosphorylated extracellular signal-regulated kinase. MnSOD2: Manganese-dependent superoxide dismutase 2. HF: heart failure. LAD: Left Anterior Descending artery. * means the suppression of FOXO3 for cardiac protection.

**Table 3:** Plant extracts modulating FOXO3 in HF.

No.	Plant Extracts	Cell Model	Dose, Time	Animal Model	Dose, Time	Mechanism	Year, Ref.
1	White Wine *	N/A	N/A	LAD ligation rats	6.5 mL/kg, 30 days	Reduction in apoptosis Increased in pAkt, pFOXO3, and peNOS expression	2008, [75]
2	Panax notoginseng	N/A	N/A	24-month-old rats	60 mg/kg, 24 weeks	Reduction in apoptosis Inhibition of oxidative damage Increase in FOXO3 and MnSOD2 expression	2018, [81]
3	Sanwei-Tanxiang *	N/A	N/A	LAD ligation rats	Fructus Choerospondiatis 360 mg/kg, Nutmeg 108 mg/kg, and Santalum album L. 54 mg/kg	Reduction in apoptosis Increased in pAkt and pFOXO3 expression	2021, [76]
4	Blueberry *	H9c2 rat cardiomyocytes	50 μg/mL, 24 h	N/A	N/A	Reduction in apoptosis Increased in pAkt and pFOXO3, and MnSOD2 expression	2022, [77]
5	Gentianella acuta	N/A	N/A	TAC-induced HF rats	600 mg/kg, 4 weeks	Reduction in autophagy Decreased in pPI3K, pAkt and pFOXO3 expression	2022, [79]
6	Polygonum cuspidatum	H9c2 rat cardiomyocytes	100 μg/mL, 24 h	Angiotensin II-induced HF mice	200 mg/kg, 4 weeks	Increased in FOXO3 and Cu/ZnSOD1 expression	2022, [82]
7	Linggui Zhugan	H9c2 rat cardiomyocytes	10 mg/mL, 24 h	Doxorubicin-induced HF mice	20 g/kg, 30 days	Reduction in apoptosis Inhibition of oxidative damage Increase in pAMPK and pFOXO3 expression	2025, [80]
8	Qili qiangxin *	H9c2 rat cardiomyocytes	100 μg/mL, 24 h	LAD ligation mice	1 g/kg, 1 week	Reduction in apoptosis Reduction in autophagy Increased in pPI3K and pAkt expression Decrease in FOXO3 expression	2025, [78]

N/A: Not available. pFOXO3: Phosphorylated forkhead box O3. pPI3K: Phosphorylated phosphoinositide 3-kinase. pAkt: Phosphorylated protein kinase B. pAMPK: Phosphorylated AMP-activated protein kinase. MnSOD2: Manganese-dependent superoxide dismutase 2. Cu/ZnSOD1: Copper/Zinc Superoxide Dismutase 1. peNOS: Phosphorylated endothelial nitric oxide synthase. HF: heart failure. LAD: Left Anterior descending artery. TAC: Transverse aortic constriction. * means the suppression of FOXO3 for cardiac protection.

**Table 4:** Pharmaceuticals modulating FOXO3 in HF.

No.	Pharmaceuticals	Cell Model	Dose, Time	Animal Model	Dose, Time	Mechanism	Year, Ref.
1	Lovastatin	Rat primary cardiomyocytes	2 μM, 48 h	N/A	N/A	Increase in pAkt, FOXO3, and p21 expression Decrease in RhoA and ROCK1/2 activity	2007, [83]
2	Simvastatin *	Rat cardiac microvascular endothelial cells	1 μM, 24 h	N/A	N/A	Reduction in apoptosis Induction of mitophagy Inhibition of oxidative damage Increase in pAkt, p70S6K, pFOXO3, and eNOS	2014, [85]
3	Simvastatin **	H9c2 rat cardiomyocytes	10 μM, 24 h	C57BL/6 mice	5 mg/kg, 3 weeks	Induction of mitochondrial dysfunction Induction of apoptosis Induction of nuclear translocation of FOXO3 Increase in pAMPK and atrogin-1 expression	2016, [84]
4	Dapagliflozin *	H9c2 rat cardiomyocytes	5 μM, 24 h	LAD ligation rats	N/A	Reduction in apoptosis Increase in glucose metabolism Increase in STC1, HIF1α, and pJNK expression Decrease in FOXO3 expression	2025, [86]
5	Dapagliflozin *	HL-1 mouse cardiomyocytes	20 μM, 48 h	Streptozotocin-induced diabetes mice	10 mg/kg, 6 weeks	Reduction in apoptosis Increase in SIRT1 and pFOXO3 expression	2025, [87]
6	Metoprolol *	N/A	N/A	Streptozotocin-induced diabetes mice	75 mg/kg, 1 week	Increase in pAkt and pFOXO3 expression	2008, [88]
7	Entinostat	N/A	N/A	Ex vivo heart with IR from Sprague Dawley rats	10 mg/kg, 24 h	Increase in nuclear FOXO3, MnSOD2, and catalase expression	2014, [89]
8	Artesunate	H9c2 rat cardiomyocytes	10 μM, 24 h	N/A	N/A	Reduction in apoptosis Inhibition of oxidative damage Increase in SIRT1, FOXO3, and MnSOD2 expression	2024, [90]
9	Dronedarone	H9c2 rat cardiomyocytes	10 μM, 24 h	TAC-induced HF rats	90 mg/kg, 8 weeks	Reduction in cell surface area Increase in SIRT1 and FOXO3 expression Decrease in ANP and BNP expression	2024, [91]

N/A: Not available. pFOXO3: Phosphorylated forkhead box O3. AcFOXO3: Acetylated forkhead box O3. pAkt: Phosphorylated protein kinase B. p70S6K: Phosphorylated 70 kDa ribosomal protein S6 kinase. pAMPK: Phosphorylated AMP-activated protein kinase. pJNK: Phosphorylated c-Jun N-terminal kinase. MnSOD2: Manganese-dependent superoxide dismutase 2. eNOS: Endothelial nitric oxide synthase. HF: heart failure. IR: Ischemia–reperfusion. TAC: Transverse aortic constriction. LAD: Left Anterior descending artery. ANP: Artery Atrial Natriuretic Peptide. BNP: B-type Natriuretic Peptide. * means the suppression of FOXO3 for cardiac protection. ** means the activation of FOXO3 for cardiac injury.

**Table 5:** Other chemicals modulating FOXO3 in HF.

No.	Chemicals	Cell Model	Dose, Time	Animal Model	Dose, Time	Mechanism	Year, Ref.
1	Bromelain *	N/A	N/A	Ex vivo heart with IR from Sprague Dawley rats	10 mg/kg, 15 days	Reduction in apoptosis Increase in pAkt and pFOXO3 expression	2008, [92]
2	3-Methyladenine	Rat primary cardiomyocytes	2.5 mM, 24 h	Aortic Banding rats	40 mg/kg, 3 weeks	Reduction in apoptosis and autophagy Decrease in pAkt, pJNK, pFOXO3, and BNIP3 expression	2012, [101]
3	Hydrogen sulfide *	H9c2 rat cardiomyocytes	100 µM, 24 h	N/A	N/A	Reduction in apoptosis Inhibition of oxidative damage Increase in pAkt pFOXO3, and cytosolic FOXO3 expression	2016, [99]
4	Lithium chloride *	Rat primary cardiomyocytes	20 µM, 24 h	Lipopolysaccharide-induced sepsis rats	100 mg/kg, 3 days	Reduction in apoptosis Increase in β-catenin expression Decrease in FOXO3 expression	2019, [100]
5	Elabela	N/A	N/A	Streptozotocin-induced diabetes mice	4.5 mg/kg, 12 weeks	Reduction in apoptosis Increase in SIRT3 and MnSOD2 expression Decrease in AcFOXO3 expression	2021, [95]
6	β-Hydroxybutyrate	H9c2 rat cardiomyocytes	5 mM, 24 h	Lipopolysaccharide-induced sepsis mice	3 g/kg, 3 days (as β-hydroxybutyrate--1,3-butanediol)	Inhibition of oxidative damage Increase in FOXO3 and MnSOD2 expression	2022, [97]
7	VHVV peptide	N/A	N/A	Spontaneously hypertensive rats	25 mg/kg, 8 weeks	Reduction in apoptosis Increase in pAkt, AMPK, SIRT1, pFOXO3, and FOXO3 expression	2022, [94]
8	DIKTNKPVIF peptide	N/A	N/A	Spontaneously hypertensive rats	10 mg/kg, 8 weeks	Reduction in apoptosis and fibrosis Increase in pAkt, AMPK, SIRT1, and FOXO3 expression Decrease in uPA, CTGF, COL1A1, and COL3A1 expression	2022, [93]
9	Taurine	N/A	N/A	LAD ligation rats	200 mg/kg, 8 weeks	Increase in FOXO3 expression Decrease in Akt expression	2023, [96]
10	1,3-Butanediol	Rat primary cardiomyocytes	4 mM, 24 h	LAD ligation rats	100 mg/kg, 4 weeks	Reduction in apoptosis Induction of autophagy Increase in FOXO3 expression Decrease in pPI3K and pAkt expression	2026, [98]

N/A: Not available. pFOXO3: Phosphorylated forkhead box O3. AcFOXO3: Acetylated forkhead box O3. pPI3K: Phosphorylated phosphoinositide 3-kinase. pAkt: Phosphorylated protein kinase B. pJNK: Phosphorylated c-Jun N-terminal kinase. MnSOD2: Manganese-dependent superoxide dismutase 2. HF: heart failure. IR: Ischemia–reperfusion. LAD: Left Anterior descending artery. * means the suppression of FOXO3 for cardiac protection.

## Data Availability

No new data were created or analyzed in this study. Data sharing is not applicable to this article.
